# Mapping cognitive diversity in older adults through community-based digital screening via mobile devices: a cross-sectional latent class analysis

**DOI:** 10.3389/fdgth.2025.1719318

**Published:** 2025-11-25

**Authors:** Suguru Shimokihara, Kazuki Yokoyama, Keitaro Makino, Nozomu Ikeda, Kiyoji Matsuyama, Tomonori Kashiwagi, Chiaki Kawanishi, Hikaru Ihira

**Affiliations:** 1 Graduate School of Biomedical Sciences, Health Sciences, Nagasaki University, Nagasaki, Japan; 2 Department of Occupational Therapy, School of Health Sciences, Sapporo Medical University, Sapporo, Japan; 3 Faculty of Medicine, Kagoshima University, Kagoshima, Japan; 4 Center for Environmental and Health Sciences, Hokkaido University, Sapporo, Japan; 5 Medical Center for Dementia, Ebetsu City Hospital, Ebetsu, Japan; 6 Department of Neuropsychiatry, School of Medicine, Sapporo Medical University, Sapporo, Japan; 7 Department of Physical Therapy, School of Health Sciences, Sapporo Medical University, Sapporo, Japan

**Keywords:** cognitive impairment, web-based screening, latent class analysis, dementia, subjective memory complaints

## Abstract

**Introduction:**

Early detection of objective cognitive impairment is essential to delay or prevent dementia; however, traditional in-person screening often faces practical barriers, including limited accessibility and substantial personnel demands. Web-based cognitive tools are promising for scalable screening. This study aimed to identify cognitive subgroups among community-dwelling older adults using latent class analysis (LCA) based on data collected through a freely accessible web-based cognitive screening platform that enables convenient participation anytime and anywhere using older adults' own mobile devices.

**Methods:**

Between September and December 2024, adults aged ≥65 from Sapporo and Ebetsu, Japan, were recruited via newspaper insert flyers (92,290 households) and community posters. QR codes linked to the study website were optimized for various devices. After obtaining electronic consent, participants completed web-based demographic surveys and cognitive assessments of memory, attention, and processing speed. Subjective health and memory complaints were recorded. LCA identified cognitive subgroups based on performance, complaints, and sociodemographic factors.

**Results:**

Among the 528 participants (mean age = 71.2; 57% female), most reported good health (86%) and daily conversations (92%). Cognitive function was generally preserved. LCA revealed four clusters: socially isolated females with high subjective memory complaints (SMCs); cohabiting males with high SMCs; cohabiting females with high health perception and preserved cognition; and older adults with cognitive decline.

**Discussion:**

The combination of mass outreach and web-based screening is feasible and effective in identifying diverse cognitive profiles. These findings highlight the mismatch between subjective and objective cognition and the relationship between social context, supporting scalable, tailored approaches and cognitive health.

## Introduction

The increasing prevalence of cognitive decline and dementia is a major public health challenge worldwide, particularly in rapidly aging societies. Given that early intervention strategies may delay or prevent progression to dementia, the timely identification of at-risk individuals has emerged as a paramount public health priority (1, 2). However, conventional cognitive screening approaches face substantial implementation barriers, which limit their population-level effectiveness. Traditional assessments typically require in-person attendance at clinical facilities or community centers, creating significant accessibility challenges for older adults with mobility limitations, transportation difficulties, or caregiving obligations (3, 4). Furthermore, venue-based screening may introduce a systematic selection bias, as participants tend to be more socially engaged, health-conscious, and physically capable compared to the broader older adult population (5, 6), potentially underestimating the true prevalence of cognitive impairment in the community.

Advances in digital and mobile health (mHealth) technologies have provided promising opportunities for overcoming these limitations. Although smartphone and Internet use among adults aged ≥65 years were limited a decade ago, recent surveys indicate that over 60% now own smartphones and over 70% regularly use the Internet (7–10). This digital transformation has enabled the development of web-based cognitive screening tools offering scalable, cost-effective, and geographically accessible alternatives to traditional assessment methods (11–19). These platforms can be deployed across diverse populations, including underserved communities with limited access to specialized geriatric services, while allowing participants to complete assessments in familiar environments at their convenience.

However, alternative recruitment approaches that can reach broader and more diverse older adult populations are urgently required, particularly due to population aging and the growing demand for inclusive public health interventions. Community-wide, large-scale recruitment strategies, such as newspaper insert flyers, local media campaigns, and widespread poster distributions, offer promising solutions by reaching individuals who may not actively seek health information or participate in organized activities (20). Furthermore, these methods are especially valuable for reaching socially isolated, technologically underserved, or otherwise marginalized older adults, who are often underrepresented in health research. By leveraging familiar and accessible communication channels, such as printed materials in community centers, supermarkets, and local newspapers, these strategies align with the public health principles of equity and population-based outreach. Their effectiveness may be further enhanced when combined with user-friendly digital platforms that minimize technological barriers and support broader participation across varying levels of digital literacy.

Despite these advancements, widespread adoption of web-based cognitive screening among older adults remains limited. Several factors may have contributed to this discrepancy. First, disparities in digital literacy and self-efficacy persist, with many older adults lacking confidence in navigating online platforms, even when device ownership is high (21, 22). Second, concerns about privacy, data security, and the potential misuse of cognitive health information can deter participation, particularly in cultures with strong preferences for in-person medical interactions (23). Third, limitations in physical, cognitive, or sensory abilities, such as visual impairment, reduced fine motor control, or mild cognitive impairment, may hinder the independent use of digital tools despite their user-centered design features, creating usability challenges (24, 25). Incorporating privacy-preserving online assessment environments and usability-focused design features may mitigate these barriers. Classifying older adults based on a combination of socio-demographic factors, cognitive performance measured via an online platform, and subjective health perceptions may provide valuable insights into designing tailored interventions and support strategies for an increasingly digital society.

The cognitive profiles of community-dwelling older adults may exhibit considerable heterogeneity when examined in relation to their subjective health perceptions and memory concerns, suggesting that objective cognitive performance alone may not fully capture the complexity of cognitive aging in real-world contexts. Accumulating evidence indicates that subjective health status is an important determinant of cognitive functioning, with individuals reporting better perceived health and demonstrating superior performance across multiple cognitive domains (26–28). Subjective memory complaints (SMCs) are another critical dimension that may modify the cognitive profiles of older adults, even in the absence of objectively detectable impairment. Studies have consistently demonstrated that individuals who report subjective memory difficulties exhibit distinct neuropsychological patterns (29–31). Moreover, SMCs are potential early indicators of future cognitive decline, with several prospective studies demonstrating that older adults with persistent memory complaints show accelerated rates of cognitive deterioration and an increased risk of progression to mild cognitive impairment and dementia (32–34). The multifaceted interplay between cognitive performance, subjective health, and memory concerns underscores the need for thorough profiling of cognitive aging trajectories in broadly sampled older populations.

Given these methodological and public health challenges, this study aimed to address a new strategy for community-based cognitive screening by integrating large-scale recruitment strategies and web-based assessment tools. Specifically, we used community-wide newspaper flyers and poster displays to recruit a more representative sample of older adults, followed by a comprehensive digital cognitive evaluation accessible from the participants' homes. Our primary objective was to characterize the heterogeneity of cognitive aging within a broadly recruited community sample using latent class analysis (LCA).

## Methods

### Participants

Participants were recruited between September and December 2024 from Sapporo and Ebetsu in Hokkaido, Japan, using a two-pronged recruitment strategy designed to maximize population reach while ensuring methodological feasibility. First, recruitment flyers were distributed to 92,290 households through newspaper insert flyers in Hokkaido Shimbun, the most widely circulated daily newspaper in the region, providing broad geographic coverage in urban and suburban areas. Second, recruitment posters were strategically displayed in community centers located in six wards of Sapporo City (Kita, Higashi, Nishi, Shiroishi, Teine, and Atsubetsu) and three districts of Ebetsu City (Ebetsu, Oasa, and Nopporo). These locations were selected based on feasibility considerations in this study. This dual-modality approach was designed to reach individuals who primarily receive health information through traditional media channels and those who frequent community gathering spaces.

### Procedures of digital-based assessment

Recruitment flyers and posters prominently featured a quick response code (QR code; a matrix barcode that encodes information in a two-dimensional pattern) that provided direct access to the study website, eliminating barriers associated with manual URL entry and facilitating a seamless transition from recruitment materials to study participation. The website was optimized for accessibility across multiple devices, including smartphones and tablet computers, with responsive design features to accommodate varying levels of technological proficiency among the participants. Upon accessing the site, potential participants were presented with a comprehensive informed consent page that detailed the study objectives, procedures, potential risks and benefits, data handling protocols, and participants' rights, including the ability to withdraw at any time without penalty.

Following electronic informed consent, the participants were automatically directed to complete a structured demographic questionnaire that captured key sociodemographic variables, followed immediately by a web-based cognitive assessments. The assessment platform incorporated user-friendly design elements, including large fonts, high-contrast displays, and intuitive navigation features. To ensure data integrity and participant privacy, all responses were encrypted during transmission and automatically stored on a secure server upon the completion of each assessment module.

Following the completion of all the cognitive assessments, the participants received immediate feedback displayed on the screen. Based on a large normative sample of 19,000 community-dwelling older adults maintained by the National Center for Geriatrics and Gerontology, each participant's performance was evaluated using age- and sex-specific means and standard deviations. This allowed the classification of cognitive function as either within the expected range or indicative of a potential decline. In cases in which a marked decline was detected, the system provided navigational guidance, recommending follow-up at specialized medical institutions that focused on dementia diagnosis and care. All contents of the study website intended for participants, including the electronic consent page, questionnaires, task instructions, and on-screen feedback, were presented in Japanese.

One advantage of this approach is that the entire cognitive assessment can be completed free of charge using a personal device, allowing participants to manage the process independently without necessarily disclosing the results to family members. When the cognitive function is preserved, feedback may enhance confidence in daily lifestyle habits. Conversely, when early signs of decline are detected, assessment can facilitate timely awareness and prevention, thereby supporting proactive engagement in cognitive health management.

### Inclusion and exclusion criteria

**Figure 1:**
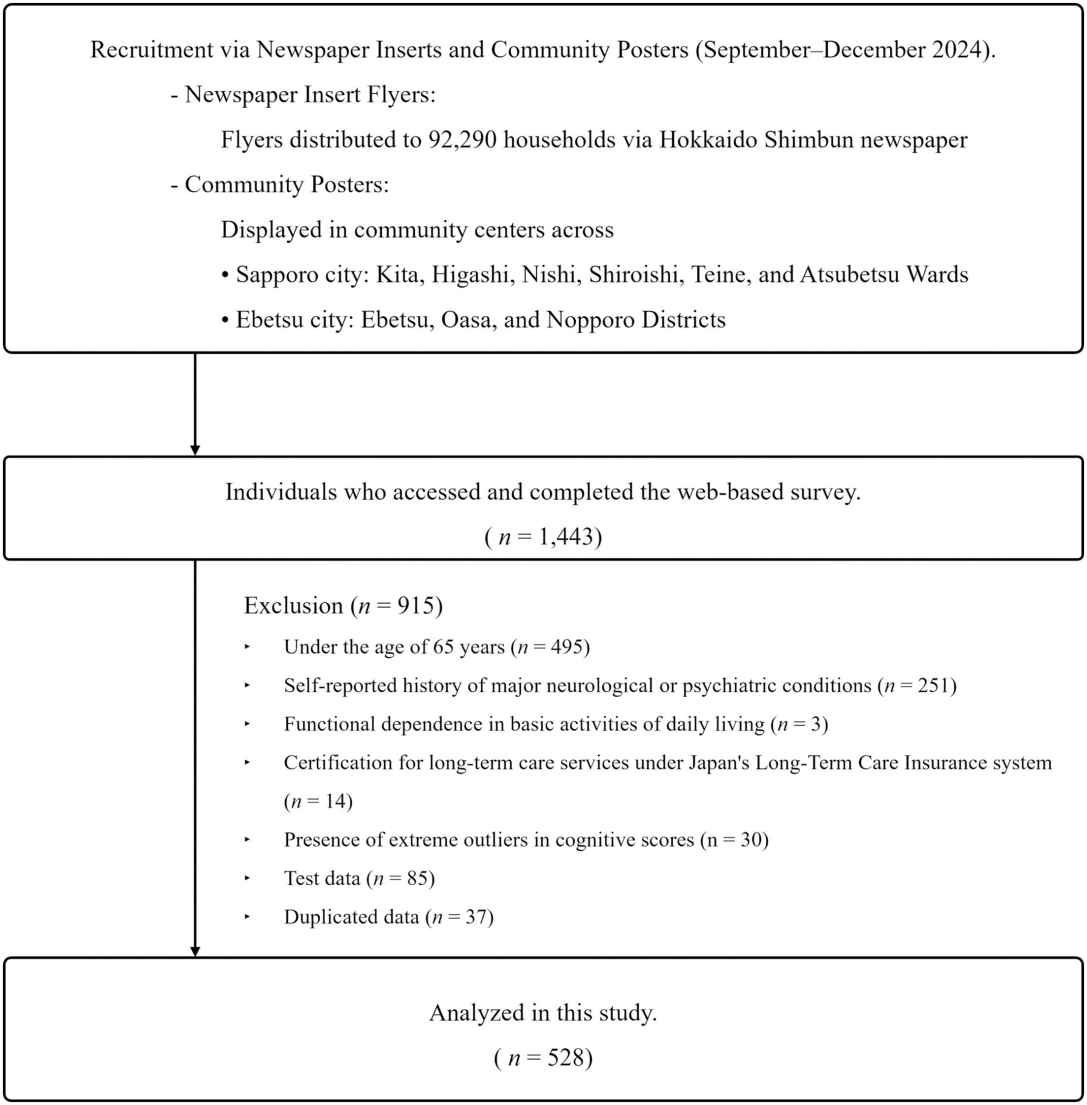
Flowchart of this study.

Participants were considered eligible for inclusion based on a comprehensive set of criteria designed to capture a representative sample of community-dwelling older adults, while ensuring data quality and analytical validity. The inclusion criteria were as follows: (1) chronological age of ≥65 years at the time of assessment; (2) absence of self-reported history of major neurological or psychiatric conditions that could impact cognitive performance, such as stroke, Parkinson's disease, clinically diagnosed depression, dementia, or mild cognitive impairment (MCI); (3) functional independence in basic activities of daily living, including eating, grooming, ambulation, bathing, and stair climbing; (4) absence of formal certification for long-term care services under the Japan's Long-Term Care Insurance system; (5) cognitive test performance within acceptable statistical bounds, defined as scores within ±3.0 standard deviations (SD) from the sample mean to exclude extreme outliers; and (6) legitimate study participation, verified by excluding registrations with usernames containing the string “Test,” which likely represented practice entries or system testing. To maintain data integrity and ensure independent observations, duplicate entries from the same individual were identified and resolved by retaining only the earliest complete record for analysis. Figure 1 shows a flowchart of the participant selection process.

### Ethical considerations

Prior to participation, all individuals were presented with an online informed consent form detailing the purpose, procedures, data handling, and voluntary nature of the study. Only those who provided electronic consent were permitted to participate in the demographic survey and cognitive assessment.

This study was approved by the Ethics Committee of Sapporo Medical University (Approval No. 6-1-20; August 20, 2024). All procedures were conducted in accordance with the ethical standards of the Institutional Research Committee and principles outlined in the Declaration of Helsinki.

### Cognitive assessment

Cognitive function was assessed using a web-based version of the National Center for Geriatrics and Gerontology-Functional Assessment Tool (NCGG-FAT). The NCGG-FAT has been validated as a reliable screening tool for detecting cognitive decline in older adults, and its utility in large-scale community settings has been demonstrated by Makizako et al. (35). The web-based version offers several advantages, including compatibility with various platforms such as smartphones and tablets, regardless of the operating system. The new assessment tool evaluates three domains: memory, attention, and processing speed. Additionally, as long as Internet access is available, the assessment can be conducted anytime and anywhere, enhancing its accessibility and scalability for both clinical and community-based applications.

#### Memory

Memory was assessed using two components: immediate recognition and delayed recall. Participants were instructed to memorize a list of 10 target words, each displayed for 2 s on a tablet screen. Thereafter, they were presented with a set of 30 words, including 10 target words and 20 distractors, and asked to identify the original words. This task was repeated three times, and the total number of correct selections across the three trials (range: 0–30) was recorded as the immediate recognition score. Approximately 20 min later, the participants were asked to recall the 10 target words. Each word correctly recalled within 60 s was awarded one point, yielding a delayed recall score (range: 0–10). Immediate recognition and delayed recall scores were evaluated for potential decline based on a cutoff of 1.0 SD below the overall participants mean score. For memory classification, the participants were categorized into one of the following three groups: robust, impairment in either immediate recognition or delayed recall, or impairment in both domains.

#### Attention

To assess attentional function, digit-span tasks were administered in forward and backward formats. The test began with two-digit sequences. After the digits were presented sequentially on the screen, the participants were instructed to input the memorized sequence by tapping the digits on the screen. In the forward digit span task, participants tapped the digits in the same order as they were presented, whereas in the backward digit span task, they tapped the digits in a reverse order. When a participant correctly responded to two trials with the same digit length, the sequence length increased by one digit. The test was terminated if the participant failed two consecutive trials of the same length. The number of correctly completed trials was recorded separately for the forward and backward tasks and used as an index of attentional capacity. Forward and backward digit span scores were evaluated for potential attentional impairment using a cutoff of 1.0 SD below the overall participant mean for each task. For attentional classification, the participants were categorized into one of three groups: robust, impairment in either the forward or backward digit span, or impairment in both domains.

#### Processing speed

Processing speed was assessed using the Symbol Digit Substitution Task. Nine symbol–number pairs were displayed at the top of the screen. A target symbol appeared at the center, and the participants selected the corresponding number from the options shown below. The score was determined by the number of correct matches completed within 90 s, with one point awarded for each correct response. In the present study, processing speed impairment was defined as a score falling 1.0 SD below the overall participant mean.

### Subjective health

Participants were asked to respond to a single item assessing subjective health status: “Do you consider yourself to be in good health?” Responses were recorded as either “Yes” or “No.” Those who responded “No” were classified as having subjective health complaints.

### SMCs

SMCs were assessed using four self-report items commonly used in geriatric cognitive screening (36, 37). Participants were asked whether they felt that they had problems with their memory; whether they found themselves forgetting where they placed things more often than before; whether they sometimes forgot the names of close friends or acquaintances; and whether others had told them that they had become forgetful. Each item was answered with either “Yes” or “No”. The number of affirmative responses was summed to generate an SMC score of 0–4, with higher scores indicating greater perceived memory difficulty.

### Sociodemographic Status

Participants provided information on their age, sex, living arrangements, and years of education. Age was categorized into six groups: 65–69, 70–74, 75–79, 80–84, 85–89, and ≥90 years. Sex was recorded as “female”, “male”, or “other/no response”. Living arrangements were assessed by asking the participants whether they lived alone or with others. As part of the sociodemographic assessment, participants were asked to respond to a single item: “Do you have conversations with someone every day?” Responses were recorded as either “Yes” or “No”. Regular social interactions, including daily conversations, are associated with better cognitive functioning in older adults (38). Besides, spontaneous everyday speech activates frontal brain regions and contributes to cognitive engagement (39). Given this evidence, daily conversation was included as a relevant variable in the cognitive health analysis. These variables were used to characterize the participants and included as covariates in the LCA to explore their associations with cognitive profiles and subjective perceptions of health and memory. Although educational level was recorded, it was not included as a classification variable in the LCA. This decision was based on the primary objective of the study, which was to identify subgroups derived from cognitive performance and key social and behavioral factors, rather than from background demographic characteristics such as education. Information on years of education was, however, used in subsequent *post-hoc* analyses to further characterize the identified clusters.

### Statistical analysis

LCA was conducted to identify distinct subgroups of participants based on their cognitive performance, SMCs, and sociodemographic characteristics. LCA is a probabilistic technique that identifies unobserved subgroups (latent classes) based on shared response patterns. It is particularly well suited to datasets comprising discrete and binary variables, and it offers advantages over traditional clustering or factor analytic methods by accommodating non-continuous data structures (40). The following variables were included in the model: age, sex, living arrangement, subjective health complaints, daily conversations, number of SMCs, memory performance, working memory performance, and processing speed. Models with two to 10 latent classes were estimated. Model fit was evaluated using the Akaike Information Criterion (AIC), Bayesian Information Criterion (BIC), likelihood ratio chi-square statistic (G²), and clinical interpretability. The optimal number of classes was determined based on the lowest AIC and BIC values as well as the clinical relevance and distinctiveness of the identified subgroups. Participants were assigned to the cluster with the highest posterior probability of membership. Descriptive statistics were used to characterize each cluster, and class labels were determined from class-specific item-response profiles and descriptive statistics. Continuous variables are presented as medians with interquartile ranges (IQRs), whereas categorical variables are reported as counts and percentages. *post-hoc* analyses were also conducted to examine differences in years of education across the identified clusters. An overall group difference assessment was performed using a Kruskal–Wallis test, followed by a Dwass–Steel–Critchlow–Fligner test to identify differences between clusters.

All analyses were conducted using R ver. 4.4.0 (R Foundation for Statistical Computing, Vienna, Austria), with LCA models implemented via the “poLCA” package ver. 1.4.1 (41). The significance level was set at *p* < .05.

## Results

### Characteristics of the participants

In total, 528 older adults were included in this study. The mean age was 71.2 years (SD = 4.8), and 57% of the participants were female. Table 1 presents the participants' characteristics. Most participants lived with others (86%) and reported good health (86%). The majority engaged in daily conversations (92%), and SMCs were distributed across the sample, with 11% reporting no complaints and 11% reporting all four items.

**Table 1:** Characteristics of the participants.

Characteristic	*n* = 528^a^
Age (years)	
65–69	225 (43%)
70–74	177 (34%)
75–79	89 (17%)
80–85	33 (6.3%)
85–89	3 (0.6%)
≥90	1 (0.2%)
Gender	
Female	299 (57%)
Male	225 (43%)
Other/no response	4 (0.8%)
Living arrangements	
Living alone	74 (14%)
Living with someone	454 (86%)
Having subjective health complaints	
Yes	76 (14%)
No	452 (86%)
Having daily conversations with someone	
Yes	486 (92%)
No	42 (8.0%)
Number of subjective memory complaints	
0	57 (11%)
1	126 (24%)
2	154 (29%)
3	131 (25%)
4	60 (11%)
Cognitive assessment	
Memory	
Robust	382 (72%)
Impairment in either immediate recognition or delayed recall	113 (21%)
Impairment in both domains	33 (6.3%)
Attention	
Robust	415 (79%)
Impairment in either forward or backward digit span	84 (16%)
Impairment in both domains	29 (5.5%)
Processing speed	
Robust	461 (87%)
Impairment	67 (13%)

a *n* (%).

Regarding cognitive performance, 72% of the participants showed no memory impairment, 21% had mild impairment in either immediate or delayed recall, and 6.3% had impairments in both. Working memory was intact in 79% of the participants, with 16% showing a mild decline and 5.5% showing impairment in both the forward and backward digit span tasks. Processing speed was preserved in 87% of the participants, whereas 13% demonstrated reduced performance.

### Identification of clusters via LCA

LCA identified a four-cluster model as the optimal solution based on model fit indices and clinical interpretability (Table 2). The four-cluster model demonstrated the lowest AIC and favorable goodness-of-fit statistics compared with models with fewer or more classes. Furthermore, clinical interpretability was supported by distinct and coherent profiles observed across the four clusters, each reflecting meaningful combinations of cognitive performance, SMCs, and sociodemographic characteristics. Each participant was assigned to the cluster with the highest posterior probability of membership.

**Table 2:** Model fit statistics for multiple models.

Cluster model	AIC	BIC	G^2^	*χ* ^2^	Notifications
2	6,630.55	6,797.04	1,168.62	31,318.70	
3	6,547.23	6,799.11	1,045.30	55,529.75	
4	6,551.68	6,888.93	1,009.75	14,310.24	
5	6,566.10	6,988.74	984.17	51,347.71	
6	6,556.07	7,064.09	934.14	7,272.50	Maximum likelihood not found
7	6,573.22	7,166.63	911.29	17,730.27	
8	6,582.04	7,260.83	880.11	4,337.20	
9	6,605.65	7,369.82	863.72	5,345.15	Maximum likelihood not found
10	6,632.24	7,481.79	850.31	4,244.05	Maximum likelihood not found

AIC, Akaike Information Criterion, BIC, Bayesian Information Criterion.

Table 3 summarizes the characteristics of each cluster after the participants were classified into the cluster with the highest posterior probability based on the LCA results. The labeling of the clusters was determined by examining the most salient sociodemographic, behavioral, and cognitive features of each cluster.

**Table 3:** Characterizing study participants via latent class modeling.

Characteristic	Cluster 1	Cluster 2	Cluster 3	Cluster 4
	*n* = 38	*n* = 258	*n* = 167	*n* = 65
% of overall participants	7.2	48.9	31.6	12.3
Age (years)				
65–69	11 (29%)	133 (52%)	81 (49%)	0 (0%)
70–74	13 (34%)	87 (34%)	66 (40%)	11 (17%)
75–79	10 (26%)	36 (14%)	18 (11%)	25 (38%)
80–85	3 (7.9%)	1 (0.4%)	2 (1.2%)	27 (42%)
85–89	0 (0%)	1 (0.4%)	0 (0%)	2 (3.1%)
≥90	1 (2.6%)	0 (0%)	0 (0%)	0 (0%)
Gender				
Female	32 (84%)	72 (28%)	167 (100%)	28 (43%)
Male	5 (13%)	183 (71%)	0 (0%)	37 (57%)
Other/no response	1 (2.6%)	3 (1.2%)	0 (0%)	0 (0%)
Living arrangements				
Living alone	38 (100%)	9 (3.5%)	19 (11%)	8 (12%)
Living with someone	0 (0%)	249 (97%)	148 (89%)	57 (88%)
Having subjective health complaints				
Yes	6 (16%)	36 (14%)	14 (8.4%)	20 (31%)
No	32 (84%)	222 (86%)	153 (92%)	45 (69%)
Having daily conversations with someone				
Yes	8 (21%)	256 (99%)	164 (98%)	58 (89%)
No	30 (79%)	2 (0.8%)	3 (1.8%)	7 (11%)
Number of subjective memory complaints				
0	5 (13%)	12 (4.7%)	35 (21%)	5 (7.7%)
1	2 (5.3%)	60 (23%)	53 (32%)	11 (17%)
2	9 (24%)	57 (22%)	68 (41%)	20 (31%)
3	17 (45%)	89 (34%)	3 (1.8%)	22 (34%)
4	5 (13%)	40 (16%)	8 (4.8%)	7 (11%)
Cognitive assessment				
Memory				
Robust	27 (71%)	173 (67%)	156 (93%)	26 (40%)
Impairment in either immediate recognition or delayed recall	9 (24%)	69 (27%)	11 (6.6%)	24 (37%)
Impairment in both domains	2 (5.3%)	16 (6.2%)	0 (0%)	15 (23%)
Attention				
Robust	29 (76%)	208 (81%)	153 (92%)	25 (38%)
Impairment in either forward or backward digit span	9 (24%)	44 (17%)	3 (1.8%)	28 (43%)
Impairment in both domains	0 (0%)	6 (2.3%)	11 (6.6%)	12 (18%)
Processing speed				
Robust	36 (95%)	258 (100%)	151 (90%)	16 (25%)
Impairment	2 (5.3%)	0 (0%)	16 (9.6%)	49 (75%)

#### Cluster 1: socially isolated females with high SMCs

This small but distinct subgroup consisted predominantly of women (84%) who lived alone (100%). Although most participants reported good self-rated health (84%), this cluster exhibited the highest prevalence of daily social isolation, with 79% not engaging in daily conversations. Notably, participants reported elevated levels of SMCs, despite showing largely preserved objective cognitive performance. A mild decline in attention was observed (24%) but did not reach the levels seen in Cluster 4.

#### Cluster 2: cohabiting males with high SMCs

The largest cluster was composed mainly of cohabiting men (71%) with high rates of daily conversation (99%) and self-reported good health (86%). Objective cognitive performance, including processing speed and memory, was relatively preserved. However, this group reported frequent SMCs (95% of participants reported one or more SMCs), suggesting a potential dissociation between perceived and actual cognitive functioning.

#### Cluster 3: cohabiting females with high subjective health and SMCs

This subgroup included only women (100%), the majority of whom lived with others (89%). Participants in this cluster demonstrated the most favorable objective cognitive scores across all domains. They also had the highest levels of self-rated health (92%) and the lowest levels of SMCs compared to the other clusters (21% of individuals in this cluster did not report any SMCs).

#### Cluster 4: older adults with cognitive decline

This cluster was characterized by the oldest age distribution, with the majority of participants aged 75 years and older (83.1%). Although most lived with others (88%), they had the lowest levels of self-rated health (69%) and the highest prevalence of processing speed impairment (75%). Memory and attention impairments were also more common in this group (60%).

A *post-hoc* analysis was conducted to examine differences in years of education across the four identified clusters. The results indicated that Cluster 3 had significantly more years of education compared to Clusters 1, 2, and 4 (*p* < .01), whereas no significant differences were observed among Clusters 1, 3, and 4. Detailed results are presented in Table 4.

**Table 4:** Results of multiple comparisons for years of education across the four clusters.

	Overall	Cluster 1	Cluster 2	Cluster 3	Cluster 4	*p*-value^a^	post-hoc^b^
	*n* = 528	*n* = 38	*n* = 258	*n* = 167	*n* = 65		
Education year	13.5 (12.0–16.0)	12.0 (12.0–14.0)	12.0 (12.0–14.0)	15.0 (12.0–16.0)	12.0 (12.0–14.0)	<.001	CL3 > CL 1, 2, 4

Median (Q1-Q3).

a Kruskal–Wallis rank sum test.

b Dwass-Steel-Critchlow-Fligner method.

## Discussion

Our findings demonstrated that web-based cognitive assessment tools can be successfully completed by a wide range of community-dwelling older adults and can hold considerable potential as an accessible and scalable approach for screening cognitive decline. Using LCA, we classified participants based on their objective cognitive performance, subjective health and memory complaints, and sociodemographic factors. The largest subgroup consisted of cohabiting males with frequent SMCs but preserved objective cognition. Another group comprised socially isolated females with high subjective complaints despite relatively intact cognitive scores. The third cluster included cohabiting females with high perceived health and good cognitive performance. The fourth group represented the oldest participants, showing marked impairments in processing speed and memory function. This study highlights diverse cognitive aging patterns and a mismatch between perceived and actual memory. Social contexts are associated with cognition and self-awareness. This recruitment method may have provided access to a representative older population.

### Characteristics of study participants in context

The participants in this study were community-dwelling older adults who voluntarily responded to large-scale recruitment via newspaper insert flyers and posters. Passive recruitment through widely distributed newspaper insert flyers and publicly posted flyers may have facilitated outreach to diverse segments of community-dwelling older adults, including those not actively engaged in formal care or research networks. The mean age was 71.2 years, with a predominance of females (57%) and a high proportion of women reporting good subjective health (86%). Most of the participants lived with others (86%) and engaged in daily conversations (92%), suggesting a relatively high level of social integration. Cognitive performance was generally preserved, with 72% of participants showing no memory impairment and 79% demonstrating intact working memory. These figures suggest that the study population included diverse older adults, including those with subtle cognitive concerns but without overt impairment. Compared to nationally representative cohorts in Japan (42–44), our sample appeared slightly younger, with fewer individuals reporting functional limitations or poor health status. Importantly, the inclusion criteria excluded individuals diagnosed with neuropsychiatric disorders or long-term care certification, which may have further shaped the sample toward relatively independent and cognitively intact individuals. Nonetheless, the diversity in SMCs and sociodemographic factors observed across the latent classes suggests meaningful heterogeneity within this seemingly healthy population.

### Interpretation of clusters

#### Cluster 1: socially isolated females with high SMCs

This small but distinct group consisted entirely of women living alone, with limited daily conversations and elevated SMCs. The overrepresentation of women aligns with prior findings that older women report more memory complaints than men even when cognitive scores are comparable (45–47). Social isolation may play a significant role in this cluster, with evidence linking limited engagement with cognitive decline and increased subjective complaints (48–52). The bi-directional relationship between isolation and depression may have exacerbated concerns (53–55). In this predominantly female cluster, heightened health consciousness may be associated with an increased frequency of SMCs. Previous research has indicated that women tend to be more attuned to their health status than men (56, 57), which may lead to a greater awareness of subtle cognitive changes and, consequently, heightened concern or anxiety regarding memory function. Clinically, this group may be overlooked by screenings that focus solely on objective performance. Therefore, healthcare professionals should consider integrating subjective assessments and sociodemographic contexts into routine evaluations to better identify at-risk individuals. Persistent subjective complaints are associated with an increased risk of future decline (32, 33), underscoring the need for multidimensional assessment and proactive support.

#### Cluster 2: cohabiting males with high SMCs

The predominance of this cluster may reflect the relatively younger age and common living arrangements of older adults, making cohabiting environments more typical in this demographic. Additionally, younger individuals may have been more likely to access web-based assessment platforms via mobile devices, contributing to their overrepresentation in this group.

This cluster consisted mainly of cohabiting older men with frequent SMCs despite intact objective cognition. This challenges prior findings that women report more memory concerns (45), suggesting that broader community samples may reveal underrecognized patterns among men. Cohabiting older men may engage in heightened self-monitoring and feel internal pressure to uphold their cognitive competence, especially after retirement (58–60). These dynamics could amplify SMCs despite intact cognition, thus underscoring gendered patterns in cognitive self-perception.

Interventions should target psychological mechanisms underlying these concerns. Psychoeducation, which normalizes age-related changes and distinguishes them from pathological changes, may reduce anxiety (61, 62). Given their intact objective cognition and relatively younger age, individuals in this cluster may benefit from health promotion strategies aimed at maintaining their current cognitive and physical functioning. Preventive interventions that reinforce self-efficacy and address subjective concerns may be particularly effective in supporting long-term well-being.

#### Cluster 3: cohabiting females with high health perception and preserved cognition

This cluster, composed of older cohabiting women with high subjective health, minimal SMCs, and good cognitive performance, may represent a resilient aging phenotype and a model for protective factors in cognitive health. Women tend to exhibit heightened awareness and engagement in health management and lifestyle practices (63), which may contribute to the preservation of cognitive function.

Beyond cognitive health, future interventions should address preventive measures against functional decline, sociodemographic adaptation to life events such as bereavement or children's departure from the household, and the management of sex-specific health concerns such as osteoporosis, which are particularly prevalent among older women.

#### Cluster 4: older adults with cognitive decline and processing speed impairment

This cluster included the oldest participants and showed the most pronounced cognitive impairment, particularly in terms of processing speed. Age-related declines in processing speed are well documented and often precede other cognitive impairments (64–66). Despite living with others and engaging in conversations, these individuals may require more intensive cognitive support and caregiver involvement. Their profile underscores the need for age-sensitive interventions and monitoring. Importantly, cohabitants may not recognize these subtle cognitive changes, especially when the decline is gradual and masked by routine interactions. This highlights the need for broader cognitive screening efforts that extend beyond clinical settings and incorporate inputs from formal and informal caregivers. A previous study showed that even among individuals who do not live alone, the rate of cognitive decline tends to be faster when cohabiting with someone other than a spouse (67). Therefore, cognitive screening from a more objective perspective, particularly by individuals outside of the family, is becoming increasingly important.

### Limitations and strengths

This study has some limitations that warrant consideration. First, its cross-sectional design precludes causal inferences regarding the relationships between cognitive performance, subjective health, and SMCs. Longitudinal follow-up is necessary to examine cognitive trajectories and transitions between clusters over time. Second, although mobile device ownership has been rapidly increasing among older adults in Japan, participants may still be biased toward relatively healthy and technologically literate individuals because participation requires access to digital devices and the ability to complete web-based assessments. As a result, individuals with lower digital literacy or more pronounced cognitive or functional decline may have been underrepresented. However, this also highlights a unique strength of the study. Because the participants predominantly consisted of digitally active older adults, the findings may reflect subtle cognitive differences that emerge before overt cognitive decline becomes evident. This interpretation is supported by recent meta-analysis showing that older adults who regularly engage with digital technologies tend to exhibit better cognitive functioning than those who do not (68). Identifying these early cognitive differences in a high-functioning, digitally connected population may offer valuable opportunities for early detection and preventive interventions. Third, although recruitment via newspaper insert flyers and posters broadened the outreach, the actual number of participants was relatively modest. One likely factor was the need to scan a QR code to access the study website, which may have posed a barrier for some older adults. This highlights a key challenge for future recruitment strategies, and future efforts should include multiple access options to promote inclusivity. Lastly, all data were self-reported or digitally assessed without clinical verification, which may have limited diagnostic precision.

Despite these limitations, this study had several strengths. This study demonstrates the feasibility of web-based cognitive screening in a community setting and introduces a novel, low-cost recruitment strategy that enhances ecological validity. The use of LCA allowed for the nuanced identification of cognitive and sociodemographic subgroups, offering insights into the diversity of ageing experiences. These findings can inform targeted interventions and public health strategies aimed at promoting cognitive wellbeing among older adults in this digitized society. Collaboration with local governments is essential for turning these findings into practical dementia prevention tools.

## Conclusions

This study identified four distinct cognitive and sociodemographic subgroups of community-dwelling older adults using LCA. By employing a web-based cognitive assessment tool, we sought to identify participants who may be inaccessible through traditional venue-based screening approaches, thereby enhancing the diversity and ecological validity of the study sample. The findings revealed that SMCs do not always align with objective cognitive performance, highlighting the need to promote accessible tools and supportive environments that enable individuals to undergo objective cognitive screening with ease. These insights underscore the importance of personalized approaches to cognitive health, particularly in community settings. Web-based screening combined with inclusive outreach strategies may serve as a scalable method for early detection and tailored support. Future research should explore the longitudinal changes within these subgroups and evaluate interventions that address both cognitive function and well-being.

## Data Availability

The raw data supporting the conclusions of this article will be made available by the authors, without undue reservation.
